# Electrospun Biodegradable Nanofibers Coated Homogenously by Cu Magnetron Sputtering Exhibit Fast Ion Release. Computational and Experimental Study

**DOI:** 10.3390/membranes11120965

**Published:** 2021-12-08

**Authors:** Anton M. Manakhov, Natalya A. Sitnikova, Alphiya R. Tsygankova, Alexander Yu. Alekseev, Lyubov S. Adamenko, Elizaveta Permyakova, Victor S. Baidyshev, Zakhar I. Popov, Lucie Blahová, Marek Eliáš, Lenka Zajíčková, Anastasiya O. Solovieva

**Affiliations:** 1 Research Institute of Clinical and Experimental Lymphology—Branch of the ICG SB RAS, 2 Timakova St., 630060 Novosibirsk, Russia; sitnikovanat9@gmail.com (N.A.S.); permyakova.elizaveta@gmail.com (E.P.); 2 Nikolaev Institute of Inorganic Chemistry SB RAS, 3 Acad. Lavrentiev Ave., 630090 Novosibirsk, Russia; alphiya@yandex.ru; 3 Research Institute of Virology, The Federal Research Center of Fundamental and Translational Medicine, 2 Timakova St., 630060 Novosibirsk, Russia; al-alexok@ngs.ru (A.Y.A.); aminisib@yandex.ru (L.S.A.); 4 Research Institute of Applied Ecology, Dagestan State University, Dahadaeva 21, 367000 Makhachkala, Russia; 5 Laboratory of Inorganic Nanomaterials, National University of Science and Technology “MISiS”, Leninsky Prospekt 4, 119071 Moscow, Russia; 6 Department of Computer Engineering and Automated Systems Software, Katanov Khakas State University, Pr. Lenin, 90, 655017 Abakan, Russia; bayd_vs@mail.ru; 7 Laboratory of Acoustic Microscopy, Emanuel Institute of Biochemical Physics RAS, Kosygina 4, 119334 Moscow, Russia; zipcool@bk.ru; 8 Central European Institute of Technology CEITEC-BUT, Purkyňova 123, 61200 Brno, Czech Republic; Lucie.Blahova@ceitec.vutbr.cz (L.B.); marek.elias@ceitec.vutbr.cz (M.E.); lenkaz@physics.muni.cz (L.Z.); 9 Department Condensed Matter Physics, Faculty of Science, Masaryk University, Kotlářská 2, 61137 Brno, Czech Republic

**Keywords:** PCL nanofibers, XPS, copper, antibacterial coating, ion release, cytotoxicity

## Abstract

Copper-coated nanofibrous materials are desirable for catalysis, electrochemistry, sensing, and biomedical use. The preparation of copper or copper-coated nanofibers can be pretty challenging, requiring many chemical steps that we eliminated in our robust approach, where for the first time, Cu was deposited by magnetron sputtering onto temperature-sensitive polymer nanofibers. For the first time, the large-scale modeling of PCL films irradiation by molecular dynamics simulation was performed and allowed to predict the ions penetration depth and tune the deposition conditions. The Cu-coated polycaprolactone (PCL) nanofibers were thoroughly characterized and tested as antibacterial agents for various Gram-positive and Gram-negative bacteria. Fast release of Cu^2+^ ions (concentration up to 3.4 µg/mL) led to significant suppression of *E. coli* and *S. aureus* colonies but was insufficient against *S*. *typhimurium* and *Ps*. *aeruginosa*. The effect of Cu layer oxidation upon contact with liquid media was investigated by X-ray photoelectron spectroscopy revealing that, after two hours, 55% of Cu atoms are in form of CuO or Cu(OH)_2_. The Cu-coated nanofibers will be great candidates for wound dressings thanks to an interesting synergistic effect: on the one hand, the rapid release of copper ions kills bacteria, while on the other hand, it stimulates the regeneration with the activation of immune cells. Indeed, copper ions are necessary for the bacteriostatic action of cells of the immune system. The reactive CO_2_/C_2_H_4_ plasma polymers deposited onto PCL-Cu nanofibers can be applied to grafting of viable proteins, peptides, or drugs, and it further explores the versatility of developed nanofibers for biomedical applications use.

## 1. Introduction

The preparation of copper/copper oxide nanofibrous materials became a prevalent topic thanks to a vast range of applications of such nanomaterials, including catalysis [1,2], disinfection [3], antiviral nanocomposites [4], antibacterial wound dressings [5,6], sensors [7], CO_2_ electrocatalytic reduction [8] and others. The advantage of using Cu nanofibers instead of other forms of nano or micromaterials is their high surface-to-volume ratio, the possibility to prepare sheets or foils of Cu nanofibers without any limitations in terms of their size, the opportunity to run the continuous roll-to-roll process, and its scalability. 

Several approaches were employed to prepare Cu-containing nanofibers: electroless deposition on a nanofibrous foil as a template [8], self-assembly method using PANI nanofibers as a template [9], incorporation of Cu by admixture of Cu nanoparticles to the electrospinning solution [10], and decoration of nanofibers with Cu nanoparticles [5]. 

The area of applications of nanofibrous mats is vast. Mainly, the biomedical use of nanofibers attracts the attention of many researchers because they efficiently accelerate wound healing [11,12], may filter bacteria and viruses [13,14], and regenerate the bones [15,16,17,18]. The use of nanofibers with Cu/Cu-oxide coatings for biomedical applications was not fully covered in the literature compared to, e.g., Ag nanoparticles. In most instances, the material was prepared using methods with high consumption of chemicals, i.e., either by soaking the nanofibers in the Cu^2+^ containing solution, the addition of Cu salts into the electrospinning solutions [19], grafting of Cu nanoparticles [20], or by admixture of Cu nanoparticles in the solution [6]. In general, authors witnessed both antibacterial and cytotoxic effects of their Cu-containing nanofibers and, interestingly, various forms of Cu (Cu^0^, Cu^+^, Cu^2+^, CuO, and Cu(OH)_2_) behave differently in different cultures.

Polyacrylonitrile (PAN) nanofibers with embedded CuO were tested for antimicrobial breathe masks with high antibacterial effects [21]. PVA nanofibers with Cu nanoparticles have shown significant inhibition zones against Gram-negative *Escherichia coli* and Gram-positive *Staphylococcus aureus* bacteria [10]. Haider et al. have shown that PLGA/CuO nanofiber scaffolds exhibited excellent antibacterial activity against *E. coli* and *S. aureus* bacterial strains [20]. The mechanism of the antibacterial action is based on the Cu^2+^ ion release. Phan and co-authors made a similar conclusion for *E. coli* and *B. subtilis* [19]. They also concluded that CuO and Cu(OH)_2_ embedded into polyacrylonitrile nanofibers would be less effective than CuSO_4_ but more efficient than metallic Cu thanks to the faster release of Cu^2+^ ions from oxidized surfaces. However, the authors did not reveal the cytotoxicity of such structures.

This work presents a facile, robust, and scalable method for preparing Cu-coated nanofibers based on magnetron sputtering of copper onto the FDA-approved biodegradable polycaprolactone (PCL) nanofibers. The temperature-sensitive polymer nanofibers have never been tested as a substrate for the Cu deposition by magnetron sputtering. The main challenge is the deposition of a well-adhered metallic coating with high Cu content PCL membrane without degradation of the nanofibrous structure. The antibacterial properties against Gram-negative and Gram-positive bacteria and cell viability of mesenchymal stromal cells were studied, and the mechanism of Cu-coated PCL nanofibers onto different strains was discussed.

## 2. Materials and Methods

### 2.1. Electrospinning of PCL Nanofibers

The electrospun nanofibers were prepared by the electrospinning of a 9 wt% solution of polycaprolactone PCL (80,000 g/mol). The processing of the sample can be found elsewhere [22]. Briefly, the granulated PCL was dissolved in a mixture of acetic acid (99%) and formic acid (98%). All compounds were purchased from Sigma Aldrich (Darmstadt, Germany). The weight ratio of acetic acid (AA) to formic acid (FA) was 2:1. The PCL solutions in AA and FA were stirred at 25 °C for 24 h. The PCL solution was electrospun with a 20 cm long wired electrode using a Nanospider™ NSLAB 500 machine (ELMARCO, Liberec, Czech Republic). The applied voltage was 50 kV. The distance between the electrodes was set to 100 mm. The as-prepared and non-treated PCL nanofibers are referred to as PCL-ref throughout the text.

### 2.2. Magnetron Sputtering

The Cu coatings were deposited by magnetron sputtering of a copper target in an ultra-high-vacuum deposition chamber (BESTEC, Germany). The input power to the magnetron was set to 37 W. Before the deposition, the chamber was evacuated down to 6.2·10^−8^ mbar. A 30 sccm flow of a high purity Ar gas (99.99%) was introduced into the deposition chamber, setting the operation pressure to 1.5·10^−3^ mbar. During the film deposition, the distance between the targets and the substrate was kept at 30 mm and the substrate holder was rotated at 10 rpm to obtain a homogenous film thickness. The deposition time was adjusted to deposit a 50 nm thick film (controlled by deposition onto Si wafer). The Cu-coated nanofibers are referred as PCL-Cu throughout the text.

### 2.3. Plasma COOH Coating

The COOH plasma polymer layers were deposited using the vacuum system UVN-2M equipped with the rotary and oil diffusion pumps. The residual pressure of the reactor was below 10^−3^ Pa. The plasma was ignited using radio frequency (RF) power supply Cito 1310-ACNA-N37A-FF (Comet, Flamatt, Switzerland) connected to the RFPG-128 disk generator (Beams & Plasmas) installed in the vacuum chamber. The duty cycle and the RF power were set to 5% and 500 W, respectively.

CO_2_ (99.995%), Ar (99.998%), and C_2_H_4_ (99.95%) were fed into the vacuum chamber. The flows of the gases were controlled using a Multi-Gas Controller 647C (MKST, Newport, RI, USA). The flow rates of Ar, CO_2_, and C_2_H_4_ were set to 50, 16.2, and 6.2 sccm, respectively. The pressure in the chamber was measured by a VMB-14 unit (Tokamak Company, Dubna, Russia) and D395-90-000 BOC Edwards controllers. The distance between RF-electrode and the substrate was set to 8 cm. The deposition time was 15 min and it led to the growth of ~100 nm thick plasma coatings. The plasma coated PCL-Cu nanofibers are referred to as PCL-Cu-COOH throughout the text.

### 2.4. Chemistry and Morphology Analysis

The microstructure of the nanofibers and the deposited plasma polymers was studied by scanning electron microscopy (SEM) using a Tescan Mira (Tescan, Brno, Czech Republic) device. The SEM micrographs were obtained in secondary emission mode with the accelerating voltage of 10 kV and working distance of 9 mm. Micrographs of 1024 × 1024 pixel were acquired. The elemental mappings were obtained using energy dispersive X-ray (EDX) detector (Oxford Instruments, High Wycombe, UK).

The chemical composition of the sample surfaces was determined by the X-ray photoelectron spectroscopy (XPS) using an Axis Supra spectrometer (Kratos Analytical, Manchester, UK) equipped with the monochromatic Al Kα X-ray source. The maximum lateral resolution of the analyzed area was 0.7 mm. The spectra were fitted using the CasaXPS software after subtracting the Shirley-type background. The binding energies (BE) for all carbon and oxygen environments were taken from the literature [22]. The BE scale was calibrated by setting the CH_x_ component at 285 eV.

### 2.5. The Ion Release Measuring

The High-Resolution Spectrometer iCAP 6500 (Thermo Fisher Scientific, Pittsburgh, PA, USA) was used. Samples size of 1 × 1 cm were placed in 5 mL of PBS and H_2_O, respectively, and incubated at 37 °C. After 1, 2, 4, and 24 h, an aliquot of 1 of 500 μL was taken. The sample solution was injected into the plasma through a nebulizer of SeaSpray type using a peristaltic pump with a rate of 0.7 mL/min. Analysis conditions: cooling argon flow—12 L/min, secondary—0.5 L/min; registration time on the first slit—15 s; on the second slit—5 s. The power supplied to the ICP inductor was 1150 W (recommended by the manufacturer of the spectrometer). The registration of emission spectra was carried out at the axial observation of plasma. In the process of sample preparation, the following reagents were used: concentrated nitric acid extra pure, 69.0–71.0% (Sigma-Aldrich, Darmstadt, Germany), deionized water purified with the Direct-Q3 system (Millipore) >18 MΩ/cm; high purity argon; single component standard solution—copper (Cu) (Merck). Samples dissolution was performed using concentrated nitric acid with heating ~ 100–150 °C. Sample preparation was performed using disposable plastic tubes with a volume of 5–15 mL, polypropylene container with a volume of 10 mL, and automatic pipette with variable volume (1.00–5.00 mL, 100–1000 μL 10–100 μL,). To determine analytes of Cu, the most intense spectral lines were used (without the spectral influence of the matrix)—328.068, 338.289; 324.754, 327.396 nm, respectively. The validation of the technique by spike experiment was provided.

### 2.6. Modeling

The classical molecular dynamics method in the LAMMPS [23] software package was applied to the irradiation simulations of PCL by Cu atoms. All interatomic interactions in the system were described by ReaxFF potentials [24]. The dimer energies were calculated by the selected potential to estimate the parameters of the interaction of copper atoms with polymer atoms (see Table 1) and comparisons were made with similar calculations by the DFT method [25,26] in the Vienna Ab initio Simulation Package (VASP) [27,28]. Despite that the ReaxFF potentials underestimate the energies of individual dimers, they qualitatively describe changes in the energy of interactions of Cu atoms with Cu, H, C, O, since the energy decreases from Cu-Cu to Cu-O both in the case of DFT and in the case of ReaxFF calculations. In addition, the difference in link lengths between DFT and ReaxFF is negligible.

### 2.7. Cell Tests

Cell viability was assayed by the MTT method and fluorescent microscopy. Human mesenchymal stromal cells were extracted from bone marrow using standard methods (the Ethics Committee approved the study of the RICEL-branch of ICG SB RAS (No 115 from 24.12.2015) and cultured in Dulbecco’s modified Eagle’s Medium (DMEM, Sigma Aldrich) that was supplemented with 10% fetal bovine serum (FBS, Gibco, Carlsbad, CA, USA). Cells were seeded in 96-well plates on scaffolds (round samples of diameter 0.5 cm) in concentration 7 × 10^3^ cells/well. Additionally, cells were cultivated in 96-well plates in a medium in which the PCL-Cu and PCL-Cu-COOH were soaked (round samples of diameter 0.5 cm in 200 µL) for 1 h and then incubated for 72 h under 5% CO_2_ atmosphere. A fresh culture medium was added to control cells. After that 5 µL of the MTT solution with the concentration of 5 mg/mL was added to each well, and the plates were incubated for 4 h and then solubilized with a dimethyl sulfoxide solution, as indicated in the manufacturer’s instructions. The optical density was measured with a plate reader Multiskan FC (Thermo Fisher Scientific, Singapore) at the wavelength of 570 nm. The experiment was repeated three times on separate days. For fluorescent microscopic analysis, cells were incubated at 37 °C in the dark with stain solution (199 cell culture media with 5 µg/mL Hoechst 33,342 and 2 µM calcein AM (Thermo Fisher Scientific, St. Louis, MO, USA) for 30 min. Live cells are determined distinguished by the presence of ubiquitous intracellular intense uniform green fluorescence.

### 2.8. Microbiology

*E. coli* ATCC25922, *S. aureus* ATCC25923, *S. Typhimurium* ATCC14028, *P. aeruginosa* ATCC27853 strains were obtained from Remel™, Thermo Fisher Scientific, USA, and Becton Dickenson, France. The bacterial strains were grown in liquid Lysogeny broth (LB) medium at 37 °C for 24 h and then were diluted in saline to give concentrations of 0.675–2.5 × 10^5^ colony-forming units (CFU) mL^−1^. Antibacterial activity against each strain was determined by the emersion of nanofibers (round samples in diameter 0.5 cm) in a medium volume of 300 µL with bacteria for 24 h. The number of viable microorganisms was estimated via counting of CFU after 24 h of cultivation. All experiments were performed in triplicate.

## 3. Results

### 3.1. Modeling of Cu Ions Penetration Depth

**Figure 1:**
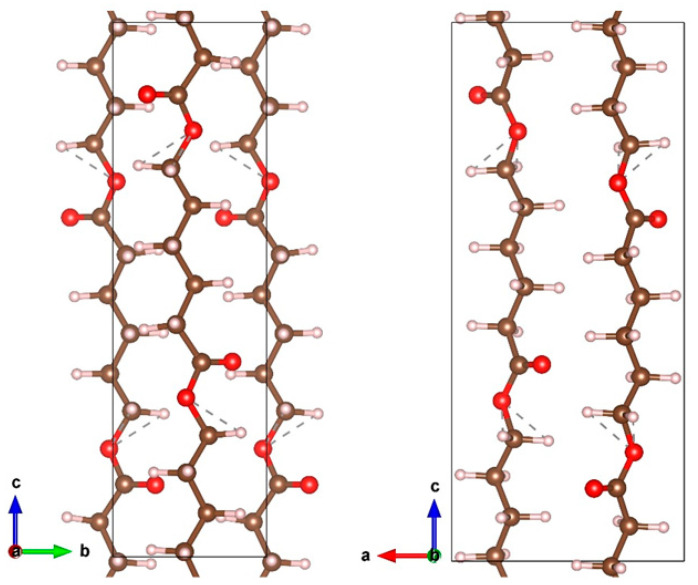
The PCL unit cell. The carbon, hydrogen and oxygen atoms are indicated by brown, gray and red colors.

In order to correctly select the conditions where no destruction of such sensitive material as PCL occurs, the computational simulation was performed before the experiments of Cu deposition. The PCL unit cell (Figure 1) was taken from [29] and relaxed in VASP, after which it was used to create a slab supercell.

To simulate the irradiation of a film of finite thickness, a PCL slab with a size of 22.7 × 3.94 × 5.31 nm, consisting of 64,800 atoms, was constructed. Periodic boundary conditions were applied in the direction of the y and z axes, and the irradiation was carried out along the x-axis. Before irradiation the slab was relaxed for 100 ps at constant pressure (NPT thermostat), then at a constant temperature (NVT thermostat) equal to T = 300 K.

**Figure 2:**
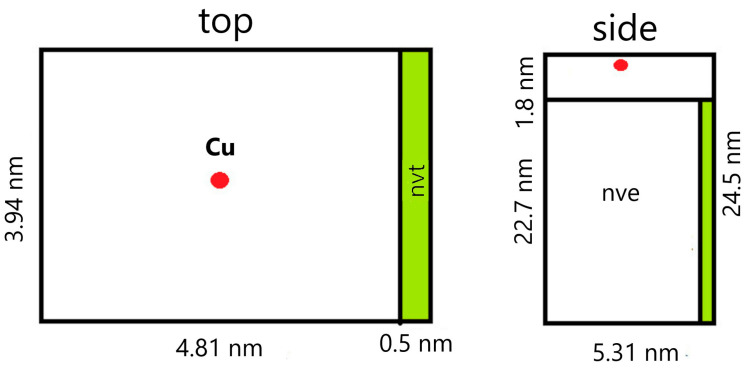
A diagram of a model with characteristic dimensions.

The metal atom was placed randomly at a distance of 1.8 nm from the PCL slab surface, mainly in the center of the YZ plane of the supercell (Figure 2). The atom was given an initial velocity component normal to the slab plane following the energy under consideration. In the simulation, a variable time step was used, which was selected from the condition that the maximum displacement of atoms did not exceed 0.001 nm. This procedure made it possible to avoid unreasonably large approaches of atoms and kept the simulation stable. For example, at the largest energies of the metal atom considered, the minimum step was 0.012 fs.

To simulate the dissipation of energy into an infinite volume of material, a 0.5 nm thick region was isolated from the side of the PCL slab to the atoms of which temperature control was applied (NVT thermostat, T = 300 K). An NVE thermostat was applied to the remaining atoms of the system, including the metal atom. The simulation continued until the energy of the metal atom exceeded the average thermal energy of the PCL slab.

**Figure 3:**
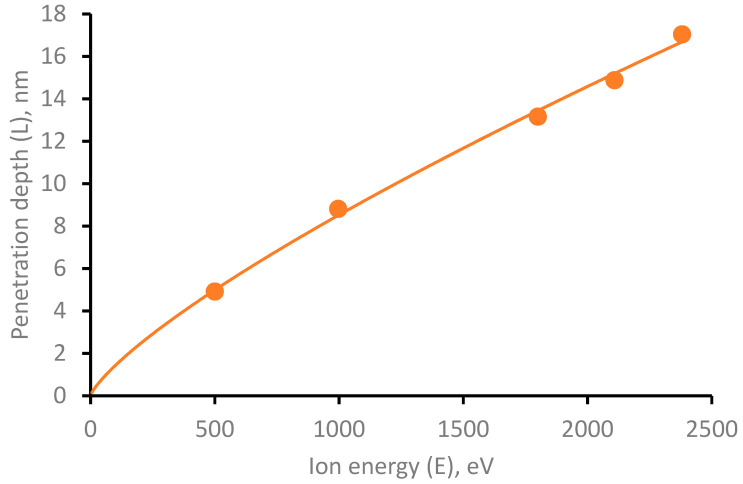
Dependence of the penetration depth of Cu atom into PCL depending on its initial energy.

The energies of a swooped Cu atom in the range from 500 eV to 2400 eV were considered. For each selected energy, a series of computer experiments were made, consisting of five simulations. The average values are shown in Figure 3.

In the energy range under consideration, a power-law dependence of the penetration depth on the atom’s energy is obtained, described by the equation L=4.07·10^−2^·E^0.7739^. It is worth noting that in the process of penetration into the PCL, the copper atom has the ability to change the movement direction due to collisions with polymer atoms. Herewith, the penetration angle of the copper atom is random, and the average deviation angle for all simulations is 5.96 degrees.

### 3.2. PCL-Cu Nanofibers

**Figure 4:**
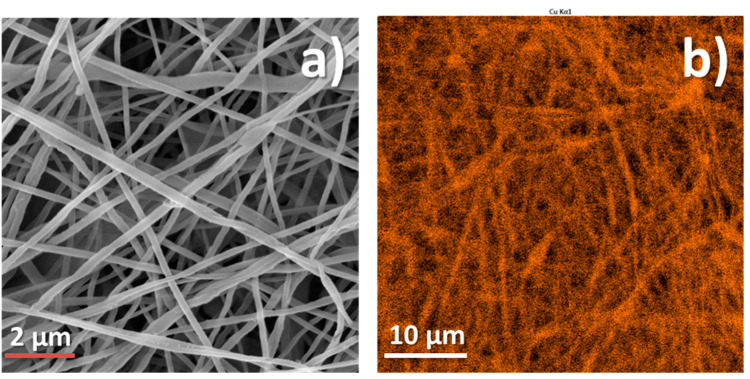
SEM micrograph (**a**) and EDS Cu signal cartography (**b**) of PCL-Cu sample.

The SEM micrograph of Cu-coated PCL nanofibrous mat is in Figure 4a. The fiber diameter was around 250 nm, and the structure of nanofibrous mesh was homogenous with no defects observed. The EDX mapping of the same sample (Figure 4b) revealed homogenous coverage of the nanofibers by copper. The XPS analysis also confirmed that the PCL-Cu sample is well covered by the copper layer. The percentages of all elements are shown in Table 2.

**Figure 5:**
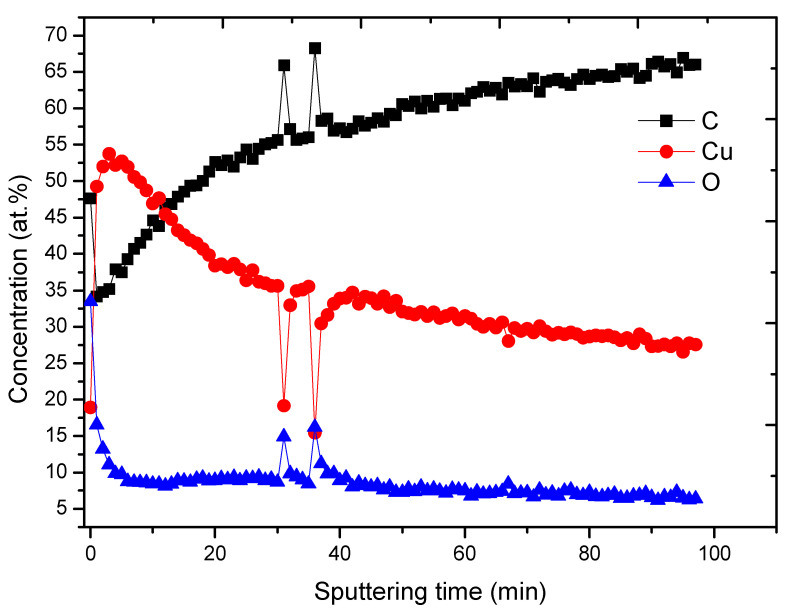
XPS depth profiling results for PCL-Cu sample.

The Cu layer covering PCL nanofibers is relatively thick, as sputtering by Ar cluster gun revealed. As shown in Figure 5, the Cu concentration increased to 53.7 at.% after the sputtering for 3 min, and the sputtering for 100 min led to its decrease to 27 at.%. Hence, a deep penetration depth of Cu ions, i.e., below 20 nm is expected as predicted by the computational analysis.

**Figure 6:**
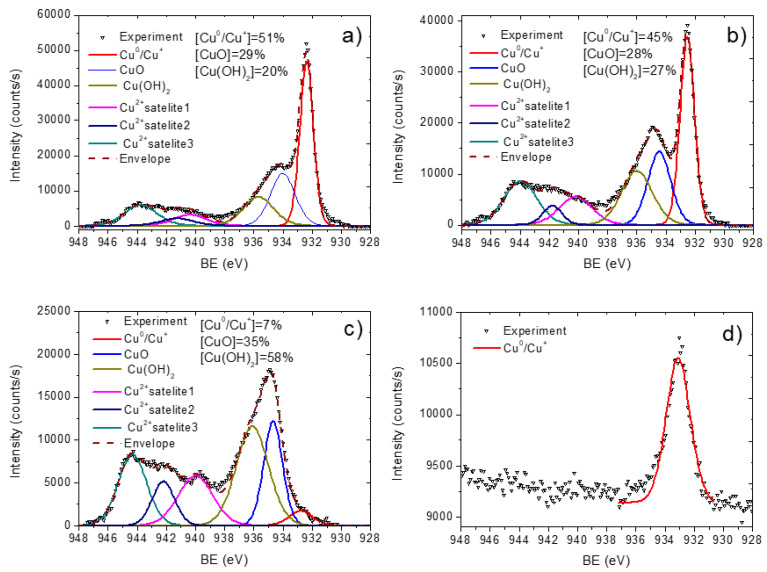
XPS Cu2p curve fitting of PCL-Cu as deposited (**a**), after 2h in PBS (**b**), after 24 h in PBS (**c**) and PCL-Cu-COOH (**d**).

**Figure 7:**
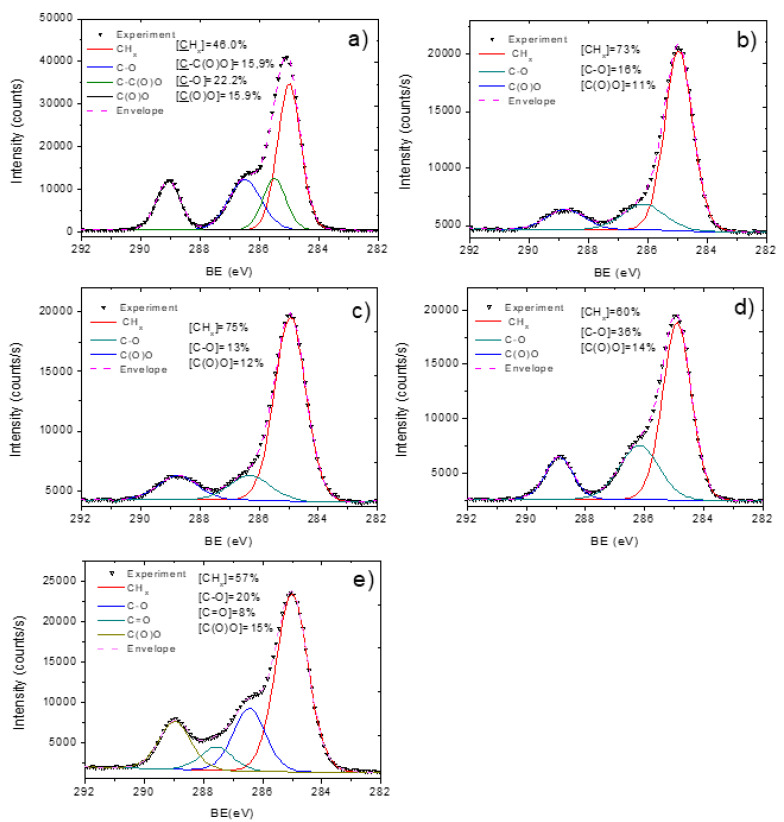
XPS C1s curve fitting of pristine PCL nanofibers PCL-ref (**a**), PCL-Cu as-deposited (**b**), PCL-Cu after two hours in PBS (**c**), PCL-Cu after 24 h in PBS (**d**) and PCL-Cu-COOH (**e**).

**Figure 8:**
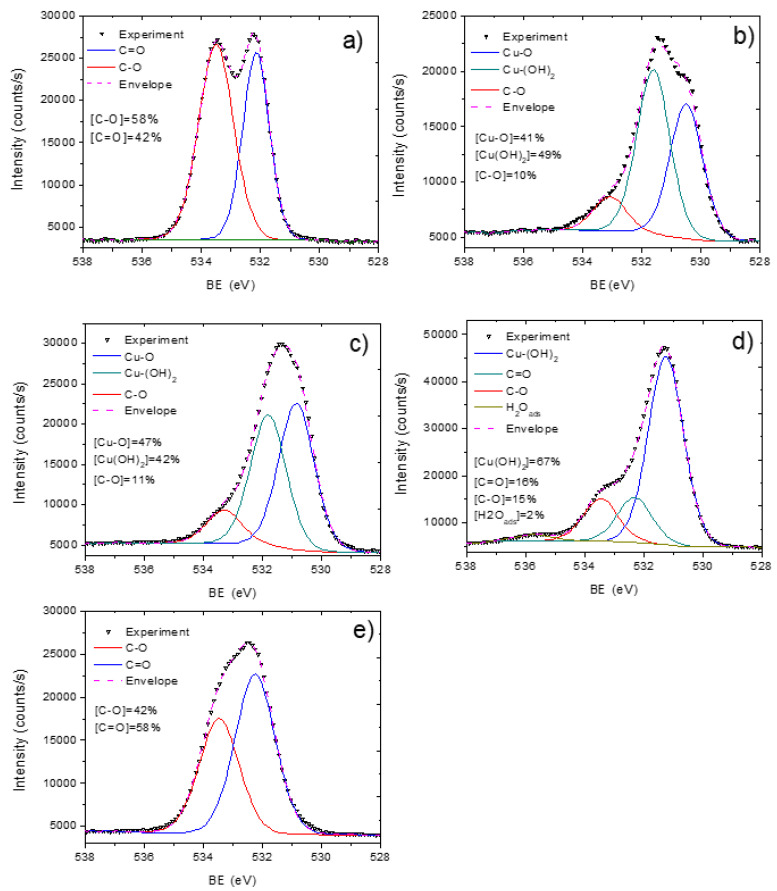
XPS O1s curve fitting of pristine PCL nanofibers PCL-ref (**a**), PCL-Cu as-deposited (**b**), PCL-Cu after two hours in PBS (**c**), PCL-Cu after 24 h in PBS (**d**), and PCL-Cu-COOH (**e**).

To investigate the chemical bonds of copper and other elements presented in the samples, the fitting of high-resolution XPS Cu2p 3/2, C1s, and O1s signals was performed (Figure 6, Figure 7 and Figure 8). We analyzed the copper environment solely by the fitting of Cu2p 3/2 signal (without the fitting of Cu2p ½) due to a higher signal of the Cu2p 3/2 line. The XPS Cu2p 3/2 signal was fitted by a sum of six peaks: metallic copper or copper oxide (I) Cu^0^/Cu^+^ (BE = 932.5 ± 0.1 eV, FWHM = 1.1 ± 0.1 eV), copper oxide (II) CuO (BE = 934.5 ± 0.1 eV, FWHM = 1.7 ± 0.2 eV), copper hydroxide Cu(OH)_2_ (BE = 935.8 ± 0.2 eV, FWHM = 1.9 ± 0.2 eV) and three Cu(II) satellites centered at 940 ± 0.2 eV, 942.2 ± 0.2 eV and 944.3 ± 0.2 eV. The BE values for all Cu2p and O1s peaks were employed from the literature: Cu^0^/Cu^+^ from [30,31], Cu(OH)_2_ from [31], and CuO 934 [32,33]. The curve fitting with percentages of each contribution is reported in Figure 6. The presence of copper oxide (II) was evident both by the high percentage of CuO peaks, high intensity of Cu(II) satellites as well by the peak of oxygen attributed to the metal oxide contribution Cu-O (BE = 530.5 eV, FWHM = 1.3 eV) in the O1s spectrum. The XPS O1s spectra also revealed peaks attributed to C-O (BE = 533.1 eV, FWHM = 1.6 eV) and OH (BE = 531.6 eV, FWHM = 1.6 eV) that most probably came from Cu(OH)_2_. Hence, the XPS O1s spectrum of PCL-Cu is completely different as compared to the spectrum of PCL-ref that was fitted by a sum of two peaks: C-O (BE = 533.4 eV) and C = O (BE = 532.2 eV), as shown in Figure 8a,b.

The shape of C1s spectra of PCL-ref and PCL-Cu additionally confirmed significant changes in the surface chemistry after Cu deposition. The XPS C1s spectrum of PCL-ref (Figure 7a) was fitted by the sum of three components, namely hydrocarbons CH_x_ (BE = 285 eV), carbon neighbored to ester group C-C(O)O (BE = 285.5 eV), ether group C-O (BE = 286.4 eV) and ester group C(O)O (BE = 289.0 eV). The FWHM for all peaks was 1.0 ± 0.1 eV. A sum of only three components fitted the XPS C1s spectrum of PCL-Cu: CH_x_, C-O and C(O)O (Figure 7b), where CH_x_ contribution with the concentration of 73% dominated over other environments. The nature of carbon presented at the surface of PCL-Cu related to the surface contaminations as well as to the signal coming from the area of nanofibers with thinner Cu coating.

### 3.3. Cu-PCL Nanofibers Coated with Plasma Polymer

The deposition of the COOH layer of PCL-Cu allows improving the wettability of PCL-Cu nanofibers and the COOH groups play a role of active sites for grafting other compounds. The successful coating was evidenced by significantly different surface composition of COOH-plasma-coated samples (PCL-Cu-COOH) compared to PCL-Cu. As shown in Table 2, the composition of PCL-Cu-COOH exhibited a deficient Cu concentration (0.4 at.%), whereas C and O percentages were similar to PCL-COOH. Additionally, the C1s and O1s curve fitting also revealed significant changes in the carbon and oxygen environments (see Figure 7e and Figure 8e). The high concentration of C(O)O environment of 15% confirmed efficient grafting of COOH moieties because C(O)O directly correlates with the concentration of carboxylic acid reactive groups [34]. The PCL-Cu-COOH was very hydrophilic, as its water contact angle decreased from 99° to 18°.

### 3.4. Stability in Water and Cu Ions Release

The antibacterial properties of PCL-Cu and PCL-Cu-COOH samples are expected to be induced by the leaching of Cu^2+^ ions. The stability of Cu layers was inspected visually and by XPS analysis of samples after soaking in PBS and by ICP-OES analyses of PBS and water after PCL-Cu and PCL-Cu-COOH soaking.

**Figure 9:**
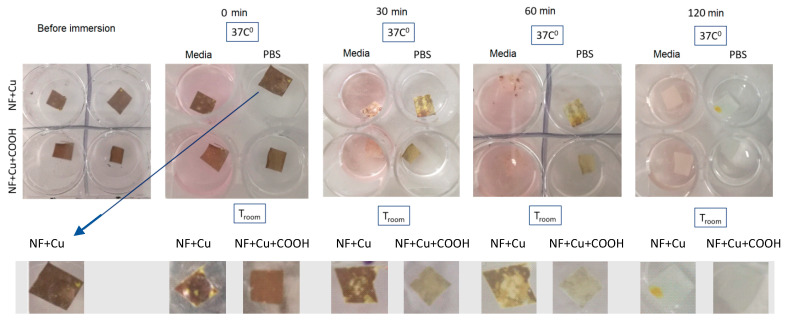
Pictures of PCL-Cu (denoted NF + Cu) and PCL-Cu-COOH (denoted as NF + Cu + COOH) after soaking in PBS and culture media.

Very rapid dissolution of Cu layer was evident even by visual inspection as shown in Figure 9. The dissolution rate depends on the liquid’s temperature and composition (culture media, PBS or deionized water). The highest dissolution rate was visually observed for PCL-Cu and PCL-COOH samples soaked in culture media at 37 °C, where no red/yellow was visible at the sample surface. It is worth noting that no flakes were floating in the liquid (Figure 9).

Nevertheless, although no coating was visible for samples after soaking in PBS, XPS analyses revealed that even after 24 h soaking, some amount of Cu still remained at the PCL-Cu-PBS24h sample (Figure 6b,c). Indeed, the oxidation and formation of hydroxide during soaking was revealed by Cu2p 3/2 curve fitting. Thus, oxidation and rapid dissolution should induce high Cu ions release.

**Figure 10:**
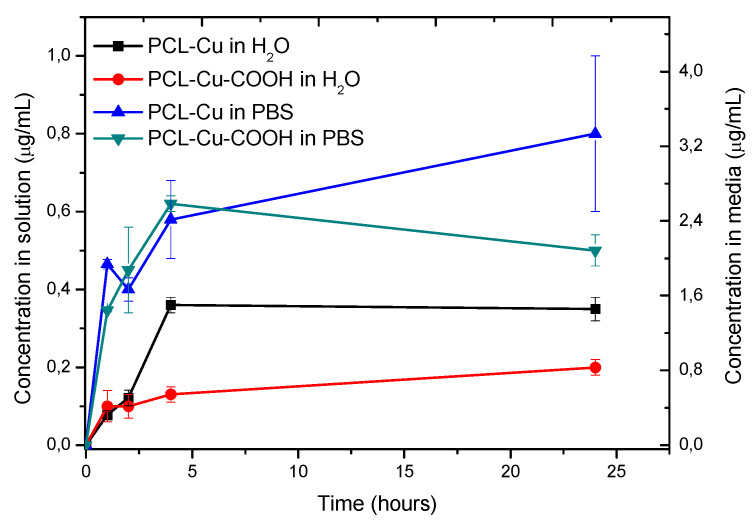
Cu ions release at 37 °C measured by ICP-OES.

As shown in Figure 10, the concentration of Cu^2+^ ions released in water or PBS at 37 °C is very high and recalculated concentration that is expected to be in the culture media while PCL-Cu cell tests (left Y-axis) exceeds 4.2 μg/mL.

### 3.5. Antibacterial Properties

**Figure 11:**
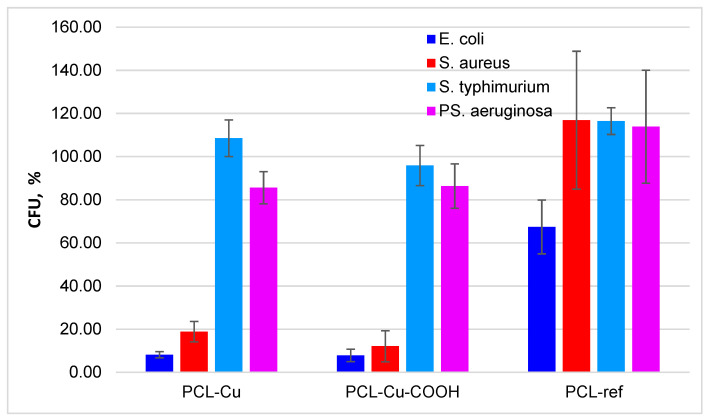
Antimicrobial effects of the PCL-ref, PCL-Cu and PCL-Cu-COOH as the percentage of colony forming units (CFU) relative to the CFU of the control.

The antimicrobial activity of nanofibers was studied on the most common pathogenic microorganisms, namely Gram-negative *E. coli, S. Typhimurium, P. aeruginosa*, and Gram-positive *S. aureus*. The PCL-ref was used as a negative control and did not have any antibacterial effect. The results are presented in Figure 11 and Table 3 as the percentage of colony-forming units (CFU) relative to the CFU of the control. The data was obtained by the emersion of nanofibers in a medium with bacteria for 24 h indicated that the PCL-Cu and PCL-Cu-COOH nanofiber reliably retard the rate of development of *E*. *coli* and *S*. *aureus*. In turn against *S*. *Typhimurium* and *P*. *aeruginosa* scaffolds had no significant antibacterial effect.

### 3.6. Cytotoxicity

**Figure 12:**
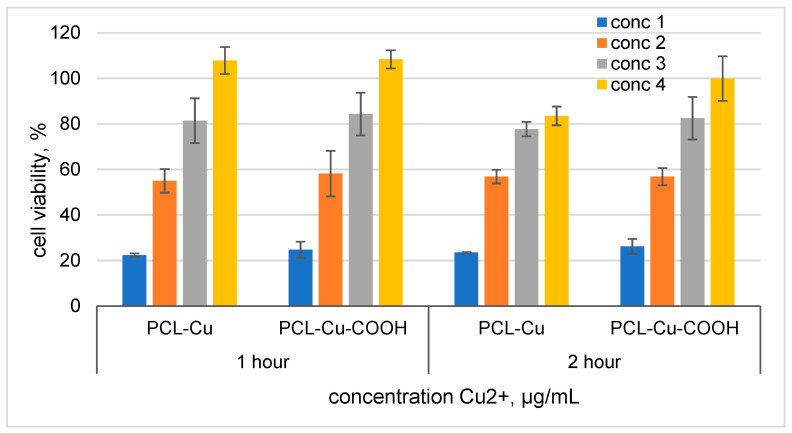
Cell viability effect of media after soaking PCL-Cu and PCL-Cu-COOH for 1 and 2 h. Cells were seeded in 96 well plates after 24 h media was replaced by medium soaked PCl-Cu and PCL-Cu-COOH. For **PCL-Cu 1** h: conc 1 = 2.3 µg/mL; conc 2 = 1.15 µg/mL, conc 3 = 0.57 µg/mL and conc 4 = 0.29 µg/mL. **PCL-Cu 2** h: conc 1 = 3.5 µg/mL; conc 2 = 1.75 µg/mL, conc 3 = 0.87 µg/mL and conc 4 = 0.44 µg/mL. For **PCL-Cu-COOH 1** h: conc 1 = 1.8 µg/mL; conc 2 = 0.9 µg/mL, conc 3 = 0.45 µg/mL and conc 4 = 0.23 µg/mL. **PCL-Cu-COOH 2** h: conc 1 = 2.9 µg/mL; conc 2 = 1.45 µg/mL, conc 3 = 0.73 µg/mL and conc 4 = 0.36 µg/mL.

**Figure 13:**
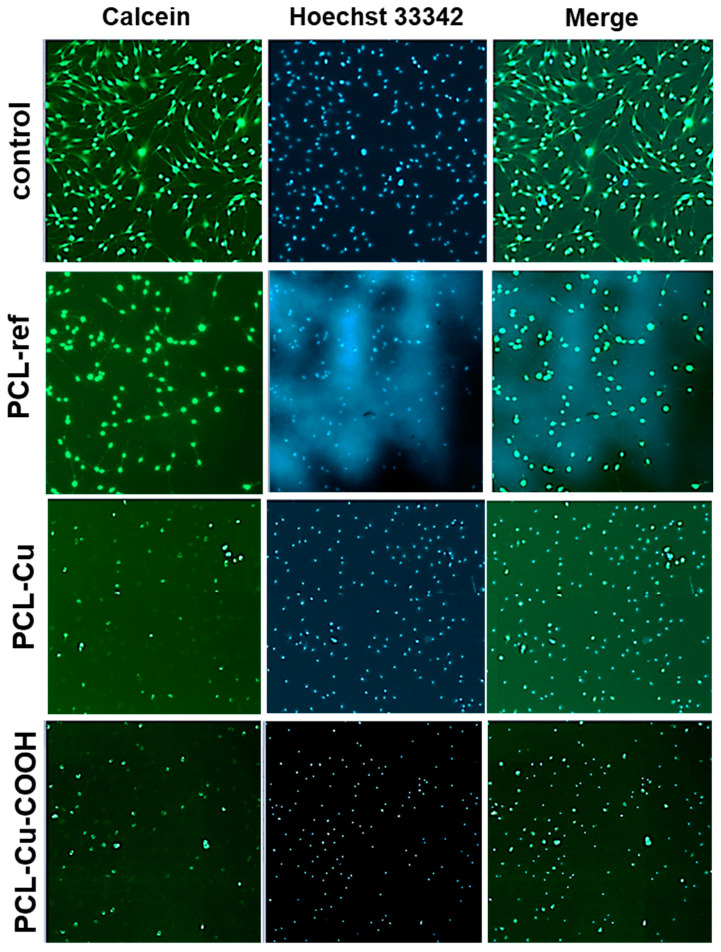
Mesenchymal stromal cells viability on the surface of PCL-ref, PLC-Cu, PCL-Cu-COOH, and on cultural plastic (control) after 3 days of cultivation. PCLs were preliminarily incubated in a culture medium for 1 h. Live cells were determined using calcein-AM, which changes from a non-fluorescent state to a fluorescent state (green) due to enzymatic conversion of intracellular esterases. The cell nuclei were stained by Hoechst 33342 (blue). Magnification 40x.

Cells seeded on PCL-Cu and PCL-Cu-COOH did not survive (data not shown). Figure 12 shows the results of the released copper ions formed during the soaking of nanomaterials in the medium effect on the MSCs viability. It has been determined that cell survival reaches 80% at copper concentrations of less than 0.87 µg/mL (Figure 12). Assessment of the viability of MSCs on nanofibers after their incubation in culture media for 1 h demonstrated an increase in their biocompatibility, the number of adhered cells on the surface of the nanomaterial was significantly higher (Figure 13). However, the functional activity of the cells, assessed with the calcein-AM fluorescent dye, was low.

## 4. Discussion

The antibacterial properties of copper have been known since the ancient period. Now, in the era of antibiotic resistance, researchers again attract much attention, including the development of Cu-based antibacterial materials. Various forms of copper are used: nanoparticles [35], Cu containing wound dressing [6,35], soluble Cu^2+^ salts, copper peroxide CuO_2_, or copper hydroxide Cu(OH)_2_. The antibacterial properties of the designed materials described in the literature differ significantly. Thus, when comparing the antibacterial action of CuO_2_ and Cu(OH)_2_, it was shown that Cu(OH)_2_ has a significantly lower antibacterial activity compared to CuO_2_ [36]. It is interesting to note that sometimes the antibacterial activity is not directly related to the concentration of copper ions. Thus, the authors demonstrated the antibacterial activity decreased with an increase of the Cu concentration [37]. In contrast, Lei et al. demonstrated that polyurethane nanofibers containing 5 wt% of CuO have no antibacterial effect and a minimum of 10 wt% of CuO is required to stimulate antibacterial effect against *E.coli* [38].

Our work demonstrated the very rapid dissolution of the Cu layer from Cu-coated PCL nanofibers and its antibacterial activity against Gram-negative *E. coli* and Gram-positive *S. aureus*. However, we did not find significant activity against the *S. typhimurium* and *Ps. aeruginosa* strains. This phenomenon is most probably related to different sensitivity towards copper for different strains. Bacteria have a number of defense mechanisms against the toxic effects of copper ions: the relative impermeability of the outer and inner membranes of the cell, which leads to a restriction of the intake (sequestration) of copper inside and the inner bacterial membranes to copper ions (intracellular sequestration), metallothionein-like proteins that absorb copper, in the cytoplasm and periplasm and energy-dependent efflux, precipitation as CuS, and extracellular complexation. Also in the cytoplasm and periplasm, some proteins actively remove copper from cells. For *S.typhimurium* and *Ps. aeruginosa* the following are known as the next copper-resistant proteins: copper detection proteins (SctR GolS, CueP for *S.typhimurium*), copper efflux proteins (GolT for *S.typhimurium* and CopA1, CopA2 for *PS. aeruginosa*) and copper sequestration proteins (CueP for *S.typhimurium*) [39,40]. The multicomponent copper efflux system CusCFBA and the multicopper oxidase CueO control the copper level and redox state in the periplasmic space, respectively [41,42]. It was shown that minimum inhibitory concentrations (MICs) of Cu against *S.typhimurium* are 8-16 times higher than for *E.coli* [42].

As a result, the MIC of *Ps. aeruginosa* to copper ions is reported at 44.8 μg/mL, significantly higher than the concentration released from nanomaterials (<10 μg/mL). Hence, possibly, the Cu^2+^ concentration leached from our PCL-Cu layers is insignificant in inducing the antibacterial effects against *S.typhimurium* and *Ps. aeruginosa.*

Our work demonstrated that largely different amounts of copper are dissolved by distilled water, phosphate buffer, and culture media. We showed that the highest dissolution rate was visually observed for PCL-Cu and PCL-COOH samples soaked in culture media. The increased rate of copper release in the nutrient medium is most likely associated with the presence of chelating compounds in it, including low-density lipoproteins, proteins (for example, ceruloplasmin), which is the carrier of copper in the body. Using various media, it is possible to control the rate of release of copper ions and, accordingly, the antibacterial effect.

Molteni et al. compared the release of copper ions in various media [41]. The authors demonstrated that the highest copper release rate was in Tris-HCl and M17 media equal 2688 and 896 μg/mL, respectively. The Cu^2+^ release in PBS and water was only 3.5 and 3.6 μg/mL, respectively. Hashmi et al. achieved Cu^2+^ concentrations up to 33.98 ppm (33.98 μg/mL) for PAN nanofibers with CuO soaked in deionized water during 72 h [21]. Hence, the media that is used for Cu^2+^ antibacterial analysis plays a significant role and affects the results.

It is worth noting that the dynamics of copper precipitation from the surface also differ. In some cases, there is a prolonged exposure for several days. In our work, similarly as in a majority of other reports [6], the rapid release of copper was observed in the first hours, followed by reaching a plateau.

Since the materials developed in our work have a great potential for biomedical use, including regenerative medicine, they must exhibit biocompatibility and activate the regenerative activity of cells. The PCL-Cu and PCL-Cu-COOH were tested for their biocompatibility towards the mesenchymal stromal cells. As mentioned earlier, the rate of copper release from the surface differs significantly depending on the medium. For MSCs cultivation we use standard culture media rich in ions, protein factors, and amino acids, which contribute to the copper extraction and binding. As a result, we have shown that seeded MSCs on PCL-Cu and PCL-Cu-COOH had a high death rate due to the high concentration of copper ions. Since the release of copper ions is fast enough, its concentration is high and cells seeded on fresh PCL-Cu/PCL-CU-COOH nanofibers have a high percentage of death. So in the reference [20], the concentration of copper released during the first 3 days is about 0.6 µg/mL, while in our work, in the first 2 h it varies from 2.9 to 3.5 µg/mL (for samples soaked in PBS). Accordingly, we experimented to assess the biocompatibility of these materials when they were previously soaked in a medium for 1 h. As a result, it was shown that cell survival increases significantly.

The influence of copper on tissue regeneration is enormous, thus the nanofibers developed in this work have excellent prospects for future research. Therefore, it will be necessary to check the effect of low pH environment, purulent wounds, with a high hydrogen peroxide content, which may increase the antibacterial activity. In this case, the high rate of copper release plays a positive role, since after cleaning the wound, Cu^2+^ concentration will decrease to the optimal level for cell regeneration. It is known that copper ions are required for collagen synthesis, stimulate VEGF production, and enhance angiogenesis [43]. Moreover, angiogenin-bound copper is a potent inducer of blood vessel development, and it binds to endothelial cell receptors and extracellular matrix components [44]. Since in our work, we also developed PCL-Cu-COOH (containing active COOH groups), which promotes strong binding of protein factors and facilitates the proliferative activity of cells [45,46]. In the future, it is planned to test the effect of the developed nanofibers on angiogenesis. It is assumed that applying platelet reach plasma (PRP) with angiogenin will reduce the toxicity of copper while having regenerative activity.

## 5. Conclusions

The Cu-coated PCL nanofibrous mats were successfully prepared by using a robust and scalable approach based on Cu magnetron sputter-deposition onto electrospun polymer nanofibers. For the first time, the large-scale modeling of PCL films irradiation by molecular dynamics simulation was performed and allowed to predict the ions penetration depth and tune the deposition conditions. The copper-coated PCL nanofibers exhibited the antibacterial effect against *E. coli* and *S. aureus* due to a fast release of Cu^2+^ ions (concentration up to 3.4 µg/mL), and sufficient biocompatibility. Thus, they may demonstrate an interesting synergistic effect when applied for wound healing. On the one hand, the rapid release of copper ions kills bacteria, while on the other hand, it stimulates the regeneration with the activation of immune cells, because copper ions are necessary for the bacteriostatic action of cells of the immune system [47]. The effect of Cu layer oxidation upon contact with liquid media was investigated by X-ray photoelectron spectroscopy revealing that, after two hours, 55% of Cu atoms are in form of CuO or Cu(OH)_2_. The reactive COOH groups available at the surface of PCL-Cu coated with carboxyl plasma polymers may be used for grafting viable proteins, peptides, or drugs. It further explores the versatility of developed nanofibers for biomedical applications use.

## Figures and Tables

**Table 1:** Dimer binding energy calculated by DFT and ReaxFF potentials, the energy difference between DFT and ReaxFF, bond length in angstroms.

Dimer	E, eV (PBE)	E, eV (ReaxFF)	ΔE, eV	R, Ǻ (PBE)	R, Ǻ (ReaxFF)
Cu-Cu	−2.75	−1.38	−1.36	2.22	2.33
Cu-H	−4.27	−2.58	−1.69	1.46	1.53
Cu-C	−4.28	−2.65	−1.63	1.76	1.61
Cu-O	−5.25	−4.05	−1.21	1.69	1.75

**Table 2:** Composition of samples (in at. %) derived from XPS analysis.

Sample Name	Cu	O	C
PCL-ref	0.0	26.1	73.9
PCL-Cu	20.4	29.0	50.6
PCL-Cu-PBS-2h	17.2	35.7	47.1
PCL-Cu-PBS-24h	8.6	43.5	47.9
PCL-COOH	0.0	27.5	72.5
PCL-Cu-COOH	0.4	26.5	73.1

**Table 3:** Antibacterial activity. The percentage is of CFU relative to the CFU of the control. The reliability of the differences was calculated according to the Student’s criterion with the degree of freedom number 4 (3 + 3-2), (*p* < 0.01). Positive control was performed using PCL-COOH with covalent bonded of Penicillin-Streptomycin-Neomycin (PSN) Antibiotic Mixture under the same conditions.

	Sample Name	PCL-Cu	PCL-Cu-COOH	PCL-Ref	Control
Microorganism					
*E. coli* ATCC25922		7.9 ± 2.9	8.1 ± 1.4	67.4 ± 12.5	100 ± 7.9
*S. aureus* ATCC25923		12.1 ± 7.2	18.8 ± 4.8	146.9 ± 92.0	100 ± 34.6
*S. typhimurium* ATCC14028		95.9 ± 9.3	108.5 ± 8.5	116.4 ± 6.2	100 ± 8.1
*PS. aeruginosa* ATCC27853		86.3 ± 10.3	85.6 ± 7.5	143.9 ± 76.2	100 ± 40.7

Gray—differences are significant compared to control.

## Data Availability

Not applicable.
